# Oo-No: *Ophidiomyces ophidiicola*-bacterial interactions and the role of skin lipids in development of ophidiomycosis

**DOI:** 10.1371/journal.ppat.1013875

**Published:** 2026-01-23

**Authors:** Jason W. Dallas, Mitra Ghotbi, Alexander J. Rurik, Tia King, Ross T. Rubin, Chloe Cummins, N. Reed Alexander, Tatyana A. Martinez, Ian B. Wilson, Emily Foster, Misael Avalos Madera, Johanna E. Crick, Donald M. Walker

**Affiliations:** 1 Department of Biology, Middle Tennessee State University, Murfreesboro, Tennessee, United States of America; 2 Institute for Plant Nutrition and Soil Science, Kiel University, Kiel, Germany; 3 Sanford School of Medicine, University of South Dakota, Vermillion, South Dakota, United States of America; University of Maryland, Baltimore, UNITED STATES OF AMERICA

## Introduction

Emerging fungal pathogens pose a great risk to global biodiversity [1], and understanding how these pathogens interact with the host and their microbiomes could aid in disease mitigation and conservation efforts. In particular, fungal pathogens of the skin are prominent threats to population health. From *Trichophyton indotineae* in humans [2], *Pseudogymnoascus destructans* in bats [3,4], and *Batrachochytrium dendrobatidis* in amphibians [5,6], fungal pathogens of the skin can have devastating effects on the host. The skin also harbors a complex microbiome that offers some degree of resistance against fungal pathogens [7,8], indicating its unique potential to mitigate the damage done by these emerging pathogens. *Ophidiomyces ophidiicola* (*Oo*), the causative agent of ophidiomycosis (commonly referred to as snake fungal disease SFD [9]), represents a unique avenue for exploring how snakes and their skin microbiomes respond to infection across multiple experimental scales (from highly controlled *in vitro* experiments to landscape-level surveys). Researchers have identified natural history patterns of snakes broadly associated with *Oo* susceptibility and behavioral alterations in afflicted individuals with potential consequences for population health [10,11]. Additionally, studies have elucidated interactions among the host microbiome, skin lipid profile, and *Oo* to identify mechanisms underpinning population-level patterns [12–15]. Snakes are highly cryptic, which makes long-term population management difficult. However, the emergence of ophidiomycosis threatens global snake populations and underpins the importance of better understanding the interactions among host skin, the microbiome, and a pathogen [16].

## Biology and ecology of *Ophidiomyces*

As an environmental saprobe, *Oo* tolerates an extensive array of environmental conditions, including a wide range of pH (5–11) and temperature (7–35 °C), and can use varying complex carbon and nitrogen sources for continued growth [17,18]. In the environment, *Oo* likely persists in the soil, including in snake hibernacula, which may serve as an environmental reservoir for the fungus [17–19]. *Oo*-infected snakes have been recorded from North America, Europe, Asia, and Australia [18], and consist of three phylogenetically distinct pathogen clades [20]. Clade I is derived from strains collected from wild European snakes. Clade II has been found on wild snakes from North America, Europe, and Asia as well as some captive populations, while Clade III largely consists of strains from captive populations distributed globally but has recently been identified in wild Asian snakes [20–22]. Multiple clonal lineages have been identified within Clade II [20,23], and the relative lack of genetic intermediates between these lineages may be indicative of several introduction events of *Oo* to North America [20]. Ultimately, the exact origin of *Oo* is unknown, primarily due to historical data deficiencies [18,20]. In North America, infections of *Oo* initially gained awareness with reports of an outbreak in an Illinois population of eastern massasauga rattlesnakes (*Sistrurus catenatus*) in 2008 [24]. However, the use of preserved museum specimens resulted in the detection of *Oo* infections dating back to 1945 in the United States [25] and 1959 in Europe [26] highlighting the usefulness of historical materials in monitoring the long-term prevalence and evolutionary diversification of *Oo*. Patterns of SFD susceptibility across snake species suggest that more aquatic species tend to show higher infection rates [27–29]. In support, fox snakes (*Pantherophis vulpinus*) in wetter environments were more likely to be infected compared to those in drier environments, suggesting habitat-associated risk factors [30]. However, these patterns are not universally consistent, and broader phylogenetic analyses indicate that ophidiomycosis susceptibility is phylogenetically and ecologically dispersed, reflecting both random and habitat-specific exposure risks [31].

## Impact of *Ophidiomyces* on snake survival, behavior, and physiology

*Oo* infections elicit an immune response which causes skin lesions that are often accompanied by a higher frequency of ecdysis in an attempt to clear the infection [9,28]. Over time, infections can progress into ophidiomycosis with lesions becoming larger and, if located on the head, may impair vision, olfaction, and infrared sensing, reducing foraging efficiency [9,32]. Evidence suggests ophidiomycosis is a chronic condition in which afflicted snakes succumb to secondary complications related to the infection rather than direct mortality by *Oo* [32,33]. Ophidiomycosis can also prompt “risky” behaviors, such as premature emergence from hibernacula and increased activity in exposed microhabitats, both of which are attempts to raise body temperature for combating infection but leave the infected snakes vulnerable to predation [28,32,34].

Physiological processes may be disrupted by *Oo.* Snakes with clinical signs exhibit lower reproductive hormone levels during critical breeding periods, negatively impacting reproductive success [35,36]. Additionally, infected snakes show an altered stress response, with elevated corticosterone levels across the year indicative of increased allostatic load [37]. *Oo* infection also increases evaporative water loss and metabolic rate, placing additional strain on the energy reserves of affected animals [38–40]. Collectively, these findings highlight that *Oo* imposes energetic costs on snakes, which may have population-level effects, although further experimental research is required to better model population responses.

## The host skin interface: Interactions between *Ophidiomyces* and skin microbiome

**Fig 1:**
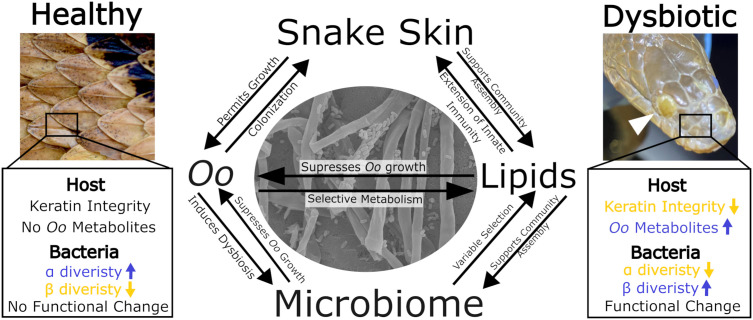
Key trends in *Ophidiomyces ophidiicola* (*Oo*) pathogen-induced dysbiosis and alteration of the snake skin environment. Blue and yellow arrows indicate increases and decreases, respectively, in host and bacterial characteristics before and after *Oo* establishment on snake skin. The shifts in host and bacterial characteristics during *Oo* colonization are modulated by bidirectional interactions among snake skin, the lipid profile, the skin microbiome, and *Oo*. The white arrow in the upper right image identifies an *Oo* lesion of an infected individual. The center image shows a scanning electron microscope image (taken by Misael Avalos Madera) of the interaction between *Oo* hyphae and bacteria *in vitro*. The healthy skin image was used with permission by Brian Miller while the image depicting dysbiosis was used with permission by Cody Godwin.

The interaction between host skin chemistry and *Oo* pathogenicity is a fundamental aspect largely ignored to date. While snake skin is composed of both alpha- and beta-keratin [41], which *Oo* can readily metabolize through a suite of enzymatic activity [42], snakes also harbor a complex lipid profile that is produced *de novo* by the skin [43]. Skin lipids aid in limiting evaporative water loss [44], intraspecific communication [45–47], and pathogen resistance [48]. Since skin lipid profiles exhibit inter- and intraspecific variation [49,50], the observed species-level differences in susceptibility to *Oo* may be underpinned in part by skin chemistry (Fig 1).

Lipids extracted from both shed and dissected skins from wild snakes have a suppressive effect on *Oo* growth despite host phylogenetic diversity and differences in natural histories [12]. This suppressive effect is likely a result of the limited capacity of the fungus to effectively metabolize high concentrations of lipids. *In vitro Oo* growth assays revealed that, even after supplementation of agar with growth-promoting keratin, common skin lipids on snakes [50–52] like oleic acid and squalene, suppressed the growth of the pathogen at high concentrations [12]. However, the presence of cholesterol in growth media did not inhibit *Oo*, illustrating the complex interplay between the most abundant sterol found in snake skin [53], different classes of skin lipids, and *Oo* growth [12].

Microbiome dysbiosis (disturbance) results in predictable changes in both the richness and structure of the snake skin microbiome and may have a negative impact on microbial function [54–56]. Similar dysbiotic trends in the microbiome were observed across two major experimental scales, including wild-caught snakes on the landscape, and live animal inoculation experiments [14,15,57]. Microbial function via metagenomic sequencing, was also shown to differ with biosynthetic gene clusters for flexirubin and fulvivirgamide unique to the microbiomes of *Oo*-negative snakes, which may aid in *Oo* inhibition [12]. *Oo*-induced shifts in the metabolic niche space are characterized as pathogen-induced dysbiosis (PID) and may have broad-reaching conservation implications if PID results in a loss of beneficial bacteria, enabling colonization of other opportunistic pathogens (Fig 1).

During pathogenic invasion of the skin, *Oo* must navigate the complex interface of skin lipids, a host immune response, and the microbiome. The host microbiome is known to be an important component in pathogen resistance [58,59] and bacterial taxa isolated from snake skin, including *Aeromonas* sp. and *Stenotrophomonas* sp., were observed to exhibit strong *Oo*-inhibitory effects *in vitro* [13]. These taxa were also identified in *Oo*-negative snakes on the landscape [14], suggesting that certain bacteria may provide a protective role against *Oo* pathogenicity through competitive exclusion, or the production of antifungal metabolites [12] that extend beyond the host’s innate and adaptive immune response. Co-culture experiments with *Chryseobacterium* sp. and *Oo* resulted in fungal growth suppression and bacterial growth facilitation suggesting that *Oo* metabolites alter the growth of certain bacterial members of the microbiome. Similarly, in spent media (cell-free supernatant) experiments, *Oo* facilitated growth of numerous taxa in the microbiome*,* and *Chryseobacterium* sp. and *Stenotrophomonas maltophilia* suppressed *Oo* growth likely due to bacterial cross-feeding [12]. The complex interactions between the skin microbiome and *Oo*, in both a direct and indirect manner, suggest resident microbes may protect the host against ophidiomycosis. Efforts to identify which bacterial-derived metabolites restrict *Oo* growth are important next steps in understanding bacterial-fungal interactions in a disease context.

## Future directions

Research progress on this relatively newly described wildlife disease has improved understanding of *Oo* biology and its impacts on snake physiology and health. Currently, there is limited research on the regional and continental distribution of *Oo* across the Global South [18]. Many of these regions have high snake biodiversity, underscoring a need for expanding ongoing surveillance efforts to understand pathogen prevalence and impact on understudied populations. Additionally, the environmental persistence and long-term reservoirs of *Oo* are understudied [19,60,61], yet are critical for monitoring and managing wildlife diseases. Linking landscape-level studies and experimental investigations of host-bacterial-fungal interactions will provide a more comprehensive understanding of how snakes respond to an emerging fungal pathogen and improve efforts in species conservation.
